# Mr. Wainwright, on an Uncommon Tumour of the Abdomen

**Published:** 1799-11

**Authors:** Thomas Wainwright

**Affiliations:** Dudley


### *To the Editors of the Medical and Phyfical Journal.*

GENTLEMEN,

I HAVE fent the following Cafe to be inferted, if you approve of it, in your very ufeful Publication.

I am, GENTLEMEN,

Your moft obliged humble fervant,

DUDLEY, *Oct.* 9, 1799. THOMAS WAINWRIGHT.

A Gentleman, in confequence of a fall from his horfe, received a violent blow on the *fcrob. cordis*; he immediately fainted, and lay for fome time fenfelefs. He walked home with great difficulty, and had nearly perifhed from the feverity of the froft. In the morning the fcrob. cordis was fwelled, tenfe, and difcoloured; the pain of it was increafed by the flighteft preffure; the pulfe was hard, fmall, and frequent; the patient could only lie on his back, was reftlefs and fleeplefs; the fkin, eyes, and urine were for feveral days tinged with bile; the pain was conftant and very acute, and after fome time extended gradually down the right fide to the bottom of the belly, and from thence to the left fide; the abdomen was now tumid, and an obfcure fluctuation was felt. A frequent dry cough came on early in the difeafe, attended with a difficulty of breathing. Eight weeks after the accident, death kindly removed him from thefe dreadful fufferings, which he had borne with exemplary fortitude.

### DISSECTION.

When the abdomen, which was fwelled and tenfe, was laid open, a tumour of extraordinary fize and appearance prefented itfelf. It occupied the whole of the hypogaftric, and part of the umbilical regions. The tumour was found to arife from a fac containing three or four gallons (by computation) of a grafs-green coloured fluid, which was evidently mixed largely with bile, for it ftained our hands and the linen, &c. yellow. This fac commenced from the inferior furface of the liver, liver, cloſe to the *ductus hepaticus*, which duct was enlarged in its courſe into the ſubſtance of the liver to the diameter of two-thirds of an inch. From this origin the ſac was traced under the diaphragm; the fluid contained having diſſected the pleura from the ribs, and formed a tumour which compreſſed the right lobe of the lungs; thence deſcending in the reverſe courſe of the aſcending colon, and puſhing the caput cœcum coli obliquely inwards, the ſac paſſed into and completely filled the pelvis, preſſing the urinary bladder into a very contracted ſpace. Aſcending from the pelvis, the ſac followed the reverſe courſe of the ſigmoid flexure of the colon to the diaphragm, where it terminated. The right and left diviſions of the ſac communicated only in the pelvis, the ſpine of the back forming a barrier between them.

The ſack was formed by a ſeparation of the peritonæum from its adheſions to the poſterior part of the pelvis, and to the pſoas and intercoſtal muſcles, and to the ribs on each ſide; the ſeparated membrane being the anterior portion of the ſac, and the cellular ſubſtance to which it had adhered the poſterior. This was moſt probably effected by bile eſcaping from a rupture either of the hepatic duct, or of the *pori biliarii*, (the external covering which theſe parts receive from the peritonæum at the ſame time not being divided) and diſſecting its way behind the peritonæum, the ſeparation would be gradually made, and the ſack conſequently produced.

It is to be remarked, that the blow was received on the ſcrobiculus cordis, and this was alſo the ſeat of the pain at the commencement of the diſeaſe. The tenderneſs and deranged ſituation of the viſcera, from the preſſure and ſize of the tumour, rendered it impoſſible to detect any rupture which might have taken place either in the hepatic duct, or in the ſubſtance of the liver.

Inflammation had extended very generally throughout the cavity. The *omentum* adhered to the ſac and to the liver. The inteſtines adhered very ſtrongly to each other and to the peritonæum in contact. The inteſtines and peritonæum were covered with a thick layer of coagulated lymph of a dirty yellow colour, and in ſome parts were black and very eaſily torn. This ſtate is perhaps the neareſt the inteſtines approach to gangrene. The ſubſtance of the liver did not appear to be much diſeaſed; it was not harder, but was perhaps of a deeper red colour, and fuller of blood than in a natural ſtate. The coat of the ſuperior ſurface of the liver was thickened, and adhered to the peritonæum. The membrane covering the inferior ſurface of the liver was thickened, thickened, and much diſeaſed and diſcoloured to the extent of two inches diameter, cloſe to the ductus hepaticus, and formed indeed a portion of the ſac. Near the hepatic duct, and in the ſubſtance of the liver, were found fragments of bile in a ſolid ſtate, and of a dark green colour, weighing perhaps from three to ſix grains. The gall bladder was contracted, and contained a ſmall quantity of light yellow-coloured bile. The kidneys, ſpleen, and pancreas were not diſeaſed.

